# Effects of particle size and lipid form of corn on energy and nutrient digestibility in diets for growing pigs

**DOI:** 10.5713/ajas.19.0196

**Published:** 2019-07-01

**Authors:** Zhiqian Lyu, Lu Wang, Yifan Wu, Chengfei Huang

**Affiliations:** 1 State Key Laboratory of Animal Nutrition, College of Animal Science and Technology, China Agricultural University, Beijing 100193, China

**Keywords:** Corn, Energy, Lipid Form, Nutrient Digestibility, Particle Size, Pig

## Abstract

**Objective:**

Two experiments were conducted to evaluate the effects of corn particle size and lipid form on the apparent total tract digestibility (ATTD) of energy and nutrients in diets for growing pigs.

**Methods:**

In Exp. 1, thirty barrows (initial body weight [BW], 53.1±3.9 kg) were allotted to 1 of 5 diets formulated with 96.9% corn ground to 441, 543, 618, 659, and 768 μm, respectively. In Exp. 2, thirty-six barrows (initial BW, 54.7±3.6 kg) were allotted to 1 of 6 diets formulated by including 2% or 15% corn germ (CG 2 or CG 15), 1% or 6% corn oil (CO 1 or CO 6), 1% CO+2% corn germ meal (CO 1+CGM 2), or 6% CO+15% corn germ meal (CO 6+CGM 15), respectively.

**Results:**

The ATTD of gross energy (GE) and the digestible energy (DE) in diet and corn grain linearly decreased as the corn particle size increased (p<0.05) from 441 to 768 μm. Particle size had a quadratic effect (p<0.05) on the ATTD of neutral detergent fiber and acid detergent fiber in diets, and which firstly increased and then decreased as the corn particle size increased from 441 to 618 μm and 618 to 768 μm, respectively. The ATTD of GE, ether extract (EE), and the DE in CO 1 diet and CO 6 diet was greater (p<0.05) than that in CG 2 diet and CG 15 diet, respectively. The ATTD of EE in CO 6 diet and CO 6+CGM 15 diet was greater (p<0.05) than that in CO 1 diet and CO 1+CGM 2 diet.

**Conclusion:**

Less than 618 μm was recommended for corn particle size in growing pig’s diet and extracted lipid had greater digestibility than the intact lipid in corn. Higher concentration of extracted CO had greater digestibility of EE compared with lower concentrations of CO diet.

## INTRODUCTION

Corn is the most important energy source in animal feed, and normally around 70% of which is used in the diet of pigs. Many factors can affect the energy value of corn for pigs, such as the variety, the grown environment, storage duration, drying method, and the breed and stages of pigs [1–3]. In addition, corn grain is typically processed before incorporating into diets for pigs. Grinding is the first but a necessary step in the manufacturing process of corn in which particle size is reduced. It has been reported that a reduction in the particle size of corn, wheat, sorghum, or soybean meal in diets improved the growth performance of pigs [4–7]. The optimal particle size (OPS) of feed ingredients will enable optimal nutrient utilization [8] and which is affected by the grain type and the body weight (BW) of pigs [9]. It has been also reported that the extract lipid as corn oil (CO), soybean oil, canola oil, and rice bran oil had greater apparent total tract digestibility (ATTD) of acid hydrolyzed ether extract (AEE) than the lipid in corn distiller’s dried grain with solubles (DDGS), full-fat soybean, press-cake, and full-fat rice bran [10–12]. The best particle size of corn for pigs has not been determined [7]. Additionally, little research has been carried on comparing the lipid digestibility in corn germ (CG) and CO, intact and extracted lipid of corn grain, respectively. Therefore, the objective of this study was to evaluate the effects of corn particle size and two lipid form of corn (CG vs CO) on the ATTD of energy, EE, and nutrient digestibility in diets fed to growing pigs.

## MATERIALS AND METHODS

This experiment followed the recommendations of “Department of China Agricultural University Animal Care and Use Ethics Committee” (AW14059102-1, Beijing, China) approved by the Institutional Animal Care and Use Committee of China Agricultural University.

### Ingredients sourcing and particle size processing

Corn grain was sourced from Fengning (Hebei province, China) and hand-harvested at 25% to 30% moisture, and then was spread on a tarp on the ground for sun-drying (Table 1). The CG, corn germ meal (CGM), and CO samples were obtained from Baoding (Hebei province, China). Corn grain was ground by a hammer mill (56×40 model, Muyang, Jiangsu, China) according to the methodology reported by Huang et al [13]. One hundred grams of corn sample was placed on top of the test sieves (sieve size 2,360, 1,180, 600, 425, 300, 150, 106 μm and a solid metal pan), and stacked from the largest to the smallest aperture size. The set of sieves positioned in a Ro-Tap sieve shaker (Tyler Industrial Products, Columbus, OH, USA) for 10 min. After sieving, the mass retained on each sieve weighed. The particle size of corn was determined by the method described by American Society of Agricultural and Biological Engineers (ANSI/ASAE S319.4; ANSI/ASAE, 2008).

### Animals, diets, and experimental design

Two studies were conducted at the Fengning Swine Research Unit of China Agricultural University (Hebei, China). In Exp. 1, thirty barrows (initial BW, 53.1±3.9 kg; Duroc×Landrace ×Large White) were allotted to 1 of 5 diets with 6 pigs per diet in a completely randomized design. The only difference among diets was the corn grain was ground to 5 specified particle sizes (i.e., 441, 543, 618, 659, and 768 μm) by a hammer sieve of 1.0, 1.5, 2.0, 2.5, and 4.0 mm, respectively (Table 2). In Exp. 2, thirty-six barrows (initial BW, 54.7±3.6 kg, Duroc×Landrace ×Large White) were allotted to 1 of 6 diets with 6 pigs per diet in a completely randomized design. Six experimental diets were formulated by including 2% or 15% CG (CG 2 or CG 15), 1% or 6% CO (CO 1 or CO 6), 1% CO+2% CGM (CO 1+CGM 2), or 6% CO+15% CGM (CO 6+CGM 15), respectively (Table 3). The lipid in CG and CGM was considered as intact form and in CO was considered as oil form. Meanwhile, ether extract (EE) content in CG2 and CO1+ CGM 2 was formulated to obtain the concentration of 4% (calculated value), and in other dietary groups (CG 15, CO 6, or CO 6+CGM 15) was 8%.

Pigs were individually housed in adjustable metabolism crates (1.7×0.6×0.7 m^3^) and given free access to water during the experiment. Room temperature was controlled at 22°C ±2°C. Each experiment lasted 19 d, including 7 d of room and cage adaptation and 7 d of diet adaptation followed by 5 d of total feces collection. During the room and cage adaptation period, a standard corn-soybean meal (SBM) diet was fed to pigs and the daily amount of feed allowance was gradually increased to 4% of the BW determined at the beginning of the diet adaptation period [14]. Daily feed allowance was divided into two equally-sized meals and provided to pigs at 08:30 h and 15:30 h, respectively. The amount of feed added to the feeders was recorded at each feeding time. Orts were removed and weighed after each meal and daily feed consumption was calculated.

### Sample collection and chemical analysis

Total feces were collected according to the methods reported by Li et al [1]. Specifically, during the collection period, an adjustable tray was placed under each metabolism crate, which permitted total collection of feces. Feces were collected twice daily at 08:30 and 15:30 h, respectively from d 15 to 19. Fresh feces were stored at −18°C immediately after collected. At the end of collection, the collected feces of 5 d from each pig were weighed, dissolved, and then homogenized thoroughly. A sub-sample of 700 g of feces was dried in a forced-air oven at 65°C for 72 h. Feed refusals and spillage were collected twice daily to be dried by the same method of drying as the feces, and then weighed. The feed and fecal samples were ground through a 1-mm screen before chemical analysis.

All chemical analysis were conducted in duplicate and repeated if they differed greater than 5%. Corn, CG, and CGM were analyzed for ash by method 942.05 [15], Ca by method 968.08 [15], and P by method 946.06 [15]. Total starch was determined by the method 76¬13.01 of the American Association of Cereal Chemists (1976) using a commercial starch assay kit (STA20; Sigma¬Aldrich Corporation, St. Louis, MO, USA). Corn, CG, CGM, feed, and fecal samples were analyzed for dry matter (DM) by method 930.15 [15], crude protein (CP) by method 984.13 [15], and EE by method reported by Thiex et al [16]. The neutral detergent fiber (NDF) and acid detergent fiber (ADF) contents were determined using filter bags and fiber analyzer equipment (Fiber Analyzer, Ankom Technology, Macedon, NY, USA) following a modification of the procedure [17]. The gross energy (GE) in corn, CG, CGM, feed, and fecal samples were determined using an Isoperibol Calorimeter (Parr 6300 Calorimeter, Moline, IL, USA) with benzoic acid as a standard. Analysis of the amino acid (AA) content of corn, CG, and CGM samples were conducted according to the method reported by Li et al [18].

### Calculations

The digestible energy (DE) was calculated by the following equation: DE = (GE_in_ − GE_out_)/F_in_, where DE is the DE content in diets (MJ/kg), GE_in_ is the total GE intake (MJ), GE_out_ is the GE content in feces (MJ), and F_in_ is the total feed intake (kg). Direct method was used to calculate DE in corn [14]. The minerals and vitamins were considered to not supply any energy. In the corn diet, the DE in diet was divided by the inclusion level of corn (DM basis) in the diet to calculate the DE in corn. The ATTD of GE and nutrient were calculated using the equation: ATTD = ([F_in_ − F_out_]/F_in_)×100%, where ATTD is the apparent total tract digestibility of nutrient in diets (%), F_in_ is the total intake of nutrient (g) from d 15 to 19, and F_out_ is the total fecal output of nutrient (g) originating from the feed that was fed from d 15 to 19.

### Statistical analysis

All the data were checked for normality, and outliers were identified using the UNIVARIATE procedure of SAS 9.4 (SAS Institute, Cary, NC, USA). An outlier was identified as “First quartile − 1.5×interquartile range” or “Third quartile+1.5× interquartile range”. Then, both data sets were analyzed using the general linear model procedure of SAS (SAS Institute, USA), with pig treated as the experimental unit. In Exp. 1, the statistical model included the particle size of corn as the only fixed effect. Polynomial contrast was also conducted using the CONTRAST statement to determine linear and quadratic effect of particle size, and contrast coefficients were generated using the interactive matrix language procedure of SAS. In Exp. 2, the statistical model included the dietary treatment as the only fixed effect, one-way analysis of variance was used to separate treatment means calculated using the LSMEANS statement, and Tukey’s multiple range test was used for post hoc comparison. Statistical significance was assumed at p<0.05.

## RESULTS

The ATTD of GE and DE in the corn and diet decreased linearly as the particle size increased (p<0.05; Table 4). There were quadratic responses in ATTD of EE, NDF, and ADF (p<0.05) as pigs were fed increased particle size of corn. There was a tendency for quadratic response to particle size of corn on ATTD of CP (p = 0.09). Specially, the ATTD of EE in pigs fed corn diets increased linearly (p<0.05) and quadratically (p<0.05) with decreasing particle size. The particle size had a quadratic effect on the ATTD of NDF and ADF in diets, and which firstly increased (p<0.05) and then decreased as the corn particle size increased from 441 to 618 μm and 618 to 768 μm, respectively.

The ATTD of GE and CP in CO 1 diet was greater (p<0.05) than that in CG 2 diet and CO 1+CGM 2 diet (Table 5). The ATTD of GE in CO 6 diet was greater (p<0.05) than that in CG 15 diet and CO 6+CGM 15 diet, respectively. The ATTD of CP in CO 6 diet was greater (p<0.05) than that in CO 6+ CGM 15 diet, but no difference compared with CG 15 diet. The ATTD of EE in CO 1 diet and CO 6 diet was greater (p< 0.05) than that in CG 2 diet and CG 15 diet. The CG 2 diet and CG 15 diet had a comparable ATTD of EE. However, the ATTD of EE in CO 6 diet and CO 6+CGM 15 diet was greater (p<0.05) than that in CO 1 diet and CO 1+CGM 2 diet. The ATTD of NDF and ADF in CG 15 diet was greater (p<0.05) than that in CG 2 diet, but no differences compared with the other diets. The DE of CO 1 diet was greater (p<0.05) than that in CG 2 diet, but no difference compared with CO 1+ CGM 2 diet. The DE of CO 6 diet was greater (p<0.05) than that in CG 15 diet, and CO 6+CGM 15 diet. The ATTD of GE and CP in CO 1+CGM 2 diet were greater (p<0.05) than that in CO 6+CGM 15 diet, but the later diet had a greater (p<0.05) ATTD of EE. The CO 6 diet had a greater (p<0.05) ATTD of EE and DE value compared with CO 1 diet. The DE in diet of CG 15, CO 6, and CO 6+CGM 15 was greater (p<0.05) than that in CG 2, CO 1, and CO 1+CGM 2 diet, respectively.

## DISCUSSION

### Nutritional value of corn, corn germ, and corn germ meal

Compared with corn grain, the CG and CGM contained a higher concentration of DM, CP, NDF, ADF, ash, and P. On as-fed basis, CO contained 24.3% EE, and which was 8.6 and 6.8 times of EE in the corn and CGM, respectively. Corn grain contained the highest concentration of starch (65.29%, as-fed basis). The concentrations of all detected AA (but leucine in CG) in CG and CGM were higher than in corn grain.

Corn grain is the most widely used energy source in the swine industry. All the detected chemical contents of corn here were within the ranges of 100 corn samples reported by Li et al [1]. The CGM is a byproduct of the wet milling industry, where processed corn grain is cleaned and steeped and then the CG is extracted for food-grade CO, resulting in CGM for animal feed [3]. Therefore, the main difference between the CGM and CG was that the former contains relatively higher fiber but lower EE content. The concentration of CP, EE, and GE of CG was 1.24, 1.23, and 1.07 times higher, respectively, and 0.63 and 0.78 times lower for the ash and starch, respectively than the values in NRC [3]. For CGM, the DM, CP, NDF, ADF, starch, and ash contents were all within the ranges of 10 CGM samples [19]. However, the concentration of EE in CGM was 1.87 and 1.68 times higher than the values from Zhang et al [19] and NRC [3], respectively. The concentration of P (0.92%) in CGM was comparable to the value of 0.90% from NRC [3], but which was 2.04 times higher compared with the average of 0.45% reported by Zhang et al [19]. The concentration of Ca in CGM was 0.33 times lower than the average reported value [19]. The GE content in CGM was 1.07 and 1.06 higher than the values reported by NRC and Zhang et al [3,19], respectively and which may be mainly due to its high EE content. All the AA contents in CGM were within the ranges of 10 CGM samples [19]. The big variation of the AA contents in the detected CG compared with those values from NRC [3] may be mainly due to only one reported sample in each literature, respectively. In addition, the variety of corn grain and processing method of CGM also can affect the chemical compositions of corn, CG, and CGM [2,3].

### Effects of corn particle size on energy and nutrient digestibility

In this study, a reduction in corn particle size increased nutrient digestibility, along with the DE content of corn and diets for growing pigs since NDF and ADF digestibility decreased from 618 to 441 μm. The ATTD of CP and EE in pigs fed corn diets increased linearly and quadratically, respectively with decreasing particle size. Consequently, the DE of diet or corn increased linearly with decreasing particle size. For young, finishing, gestating, and lactating sows, a reduction of particle size from 1,000 to 400 μm also significantly improved the ATTD of dietary nutrients [20–23]. Those findings could be related to increased effect of surface area interacting with digestive enzymes [7]. The quadratic responses in ATTD of NDF and ADF might be associated with microbial fermentation of pigs. Excessively fine diets decreased abundance of fiber degradation associated bacteria, thereby affecting negatively the digestion of the fiber. It has been shown that the decrease in the particle size of wheat from 470 to 430 μm decreased the abundances of beneficial bacteria (*Bifidobacterium* sp. and *Lactobacillus* sp.), but also increased bacterial pathogens (*Eschericha coli*), which decreased significantly the diversity of gut microflora [6]. Meanwhile, the coarse diets hinder the combination of nutrients and enzymes. Specifically, the main reason for increasing in the ATTD of GE in diets as the corn particle size decreased may be due to the increased contraction to the starch granules for α-amylase, which increases starch digestibility [24]. It is already known that the starch granules are firmly encapsulated by proteins in the endosperm, which directly interferes with the digestion of total polysaccharides and the hydrolysis rate of the digestive enzymes [25]. Grinding is related to increasing the production of hydrochloric acid and increasing the activity of pepsin in the stomach, which is essential for breaking down the protein matrix around the starch granules [20,24].

### The optimal particle size of corn grain

From the current results of energy and nutrient digestibility, diet with corn ground up to 618 μm had the highest ATTD of CP, NDF, and ADF, and therefore, up to 618 μm for corn was recommended in growing pig’s diet. Lahaye [26] reported that decreasing the particle size of wheat to 500 μm increases nitrogen digestibility, but a further decrease of particle size did not result in significant improvements. However, the particle size of wheat from 430 to 470 μm was acceptable in diets for growing pigs [6]. Rojas and Stein [20] observed a linear increase in the apparent ileal digestibility of starch and GE as the corn particle size decreased from 865 to 339 μm. The OPS of 618 μm for corn observed in this study was within the wide range previously reported for all stages of pigs. However, decreasing particle size of either extruded-expelled SBM from 965 to 639 μm or solvent-extracted SBM from 1,226 to 444 μm did not affect nursery pig growth performance [4]. Those findings indicated that particle size of ingredients affects nutrient digestibility of diets, but did not necessarily affect growth performance of pigs.

Except for the above factors that we should consider in the determination of OPS, some other parameters should also be taken into account. It has been reported that pigs fed ingredients with a finely ground grain may develop gastric ulcers [21] and development of ulcers is considered one of the major reasons for economic losses in the swine industry. Vukmirović et al [8] also reported that less than 400 μm are considered undesirable due to negative influences on the gut health. Huang et al [13] revealed that finely ground corn (320 μm) in diets increased the total white blood cells compared with coarsely ground corn (452 μm), suggesting that it may exert negative effects on the inflammatory response [27]. Considering the energy and nutrient digestibility, we recommended that milling corn to 618 μm is optimal in diets for 50 kg pigs.

### Effects of corn lipid form on energy and nutrient digestibility

In this study, we mainly wanted to investigate the effect of two lipid forms of corn: extracted and intact, on the energy and nutrient digestibility in two dietary EE levels of 4% and 8%. The analyzed EE contents in the six experimental diets in Exp. 2 generally met the aimed two EE levels except that 3.39% EE (15% lower compared with 4%) in CO 1 diet was detected. The EE measured in this study referred to crude fat with acid hydrolysis before ether extraction.

In the current study, the ATTD of EE was compared in corn diets containing intact oil (CG) and extracted oil (CO). The results indicated that the ATTD of EE in a diet containing extracted oil was greater than that in a diet containing intact oil in CG, which was in good agreement with previous results of AEE digestibility [10]. Similarly, the ATTD of AEE in corn DDGS, high protein-distiller’s dried grain, high-oil corn, full-fat rice bran, full-fat soybean, and canola press-cake were lower than in their corresponding extracted oil of rice oil, soybean oil, and canola oil [11,12]. These findings demonstrated that the extracted oil in most plant-originating ingredients was greater than in intact form. The greater digestibility of extracted oil than intact oil may be due to some of the intact oil being bound or encapsulated in the cell membranes or fiber compounds in the ingredients [28]. The ATTD of GE in CO1 was greater that in CG2, which was caused by higher EE digestibility in CO1. It has been shown that energetic efficiency of lipids is greater than that of starch, protein and dietary fiber and this largely affects the ATTD of GE [11]. The ATTD of CP observed in CO1 was greater than that in CG2. It indicted that CP in CG was more difficult to be digested than that in corn used in the current study. There is a negative correlation between fiber and GE digestibility [18, 19]. When CGM was contained in CO diets, the relatively high content of fiber components leads to a decrease in ATTD of GE.

The greater DE in CG15 than that in CG2 was caused by higher GE in CG15 than CG2 with similar GE digestibility. The greater DE in CO6 than CO1 was attributed to the same reason. The lower CP digestibility in CO 6+CGM 15 diet compared with CO 1+CGM 2 diet may be mainly due to higher inclusion level of CGM in the former diet. The CGM was processed at high processing temperatures which resulted in Maillard reactions [29], thereby decreasing the digestibility of CP in CO 6+CGM 15 diet. It was reported that the excretion of NDF-binding N increased in the upper gastrointestinal tract as dietary NDF increased [30]. The findings that diets containing higher levels of extracted CO had greater digestibility of EE compared with lower levels of CO in diets was in agreement with previous results [10–12]. However, no difference was observed for the ATTD of EE between CG 2 diet and CG 15 diet, containing two levels of intact corn lipid. Meanwhile, ATTD of EE was not affected when CGM was contained in diets. Those results further demonstrated that the intact lipid in CG had low digestibility, and intact oil would not result in any EE digestibility change.

## CONCLUSION

The ATTD of GE, CP, EE, and the DE of diet and corn linearly decreased as the corn particle size increased from 441 to 768 μm. According to the current results, the OPS of corn for growing pigs was 618 μm. The extract CO had greater total tract digestibility than intact lipid in CG. In addition, a diet containing a high level of extracted CO had greater total tract digestibility compared with a diet containing a low level of extracted CO, but there were no differences for the ATTD of intact lipid between diets containing 15% and 2% CG. Further experiments on the effect of particle size on growth performance and stomach or intestine morphology needs to be carried out to investigate the minimum particle size of corn for growing pigs.

## Figures and Tables

**Table 1:** Chemical composition of corn, corn germ, and corn germ meal used in experiment 1 and 2 (%, as-fed basis)

Items	Corn	Corn germ	Corn germ meal
Proximate composition			
Dry matter	88.53	90.01	91.97
Crude protein	8.09	18.40	21.13
Ether extract	2.81	24.30	3.56
Neutral detergent fiber	9.32	19.93	44.88
Acid detergent fiber	2.33	7.66	11.27
Starch	65.29	18.32	12.31
Ash	1.36	3.53	3.64
Calcium	0.02	0.02	0.03
Total phosphorus	0.27	1.11	0.92
Gross energy (MJ/kg)	16.16	22.04	18.72
Indispensable amino acid			
Arg	0.40	1.09	1.16
His	0.26	0.45	0.71
Iso	0.26	0.41	0.71
Leu	1.13	1.09	1.82
Lys	0.25	0.79	0.87
Met	0.16	0.26	0.37
Phe	0.38	0.58	1.00
Thr	0.30	0.53	0.83
Trp	0.06	0.11	0.17
Val	0.46	0.75	1.20
Dispensable amino acid			
Ala	0.57	0.90	1.52
Asp	0.55	1.14	1.36
Cys	0.20	0.32	0.41
Glu	1.45	1.98	2.77
Gly	0.30	0.73	1.09
Pro	0.80	0.88	1.36
Ser	0.38	0.55	0.91
Tyr	0.27	0.39	0.43
Total indispensable amino acids	3.66	6.06	8.84
Total dispensable amino acids	4.52	6.89	9.85
Total amino acid	8.18	12.95	18.69

All analysis was conducted in duplicate.

**Table 2:** The ingredient and analyzed chemical compositions of experimental diets for growing pigs in experiment 1 (%, as-fed basis)

Items	Corn particle size (μm)				

	441	543	618	659	768
Ingredients					
Corn	96.9	96.9	96.9	96.9	96.9
Dicalcium phosphate	1.70	1.70	1.70	1.70	1.70
Limestone	0.60	0.60	0.60	0.60	0.60
Salt	0.30	0.30	0.30	0.30	0.30
Premix1)	0.50	0.50	0.50	0.50	0.50
Analyzed nutrient levels					
Dry matter	87.62	87.28	96.91	86.71	87.01
Crude protein	7.83	7.91	7.88	8.01	7.92
Ether extract	2.72	3.01	2.89	2.76	2.88
Neutral detergent fiber	9.02	8.89	8.94	8.88	8.94
Acid detergent fiber	2.25	2.03	2.33	2.27	2.48
Calcium	0.61	0.60	0.60	0.61	0.61
Total phosphorus	0.47	0.46	0.48	0.48	0.49
Gross energy (MJ/kg)	15.64	15.59	15.52	15.63	15.51

All analysis was conducted in duplicate.

1) Premix provided the following per kilogram of complete diet: 5,512 IU vitamin A as retinyl acetate, 2,200 IU vitamin D_3_ as cholecalciferol, 30 IU vitamin E as DL-alpha-tocopheryl acetate, 2.2 mg vitamin K_3_ as menadione nicotinamide bisulfite, 27.6 μg vitamin B_12_, 4 mg riboflavin, 14 mg pantothenic acid as DL-calcium pantothenate, 30 mg niacin, 400 mg choline chloride, 0.7 mg folacin, 1.5 mg thiamin as thiamine mononitrate, 3 mg pyridoxine as pyridoxine hydrochloride, 44 μg biotin, 40 mg Mn as MnO, 75 mg Fe as FeSO_4_·H_2_O, 75 mg Zn as ZnO, 100 mg Cu as CuSO_4_·5H_2_O, 0.3 mg I as KI, and 0.3 mg Se as Na_2_SeO_3_.

**Table 3:** Ingredient and analyzed chemical compositons of experimental diets for growing pigs in experiment 2 (%, as-fed basis)

Items	Experimental diets1)					

	CG 2	CG 15	CO 1	CO 6	CO 1+CGM 2	CO 6+CGM 15
Ingredient						
Corn	94.9	81.9	95.9	90.9	93.9	75.9
Corn oil	-	-	1.0	6.0	1.0	6.0
Corn germ	2.0	15.0	-	-	-	-
Corn germ meal	-	-	-	-	2.0	15.0
Dicalcium phosphate	1.7	1.7	1.7	1.7	1.7	1.7
Salt	0.3	0.3	0.3	0.3	0.3	0.3
Limestone	0.6	0.6	0.6	0.6	0.6	0.6
Premix2)	0.5	0.5	0.5	0.5	0.5	0.5
Analyzed nutrient levels						
Dry matter	87.72	87.52	86.64	87.36	87.04	86.78
Crude protein	7.77	8.63	7.73	7.05	7.43	8.56
Ether extract	3.97	7.35	3.39	7.91	4.13	8.76
Neutral detergent fiber	10.80	13.61	9.79	9.59	10.68	15.75
Acid detergent fiber	2.72	3.98	2.45	2.34	2.57	4.72
Calcium	0.65	0.66	0.61	0.63	0.64	0.61
Total phosphorus	0.56	1.01	0.53	0.47	0.48	0.98
Gross energy (MJ/kg)	16.12	16.95	15.98	16.84	16.08	17.17

All analysis was conducted in duplicate.

1) CG 2 and CG 15, corn germ with inclusion rate of 2% and 15% in diet, respectively; CO 1+CGM 2, corn oil with inclusion rate of 1% combined with corn germ meal with inclusion rate of 2% in diet; CO 6+CGM 15, corn oil with inclusion rate of 6% combined with corn germ meal with inclusion rate of 15% in diet; CO 1 and CO 6, corn oil with inclusion rate of 1% and 6% in diet, respectively.

2) Premix provided the following per kilogram of complete diet: 5,512 IU vitamin A as retinyl acetate, 2,200 IU vitamin D_3_ as cholecalciferol, 30 IU vitamin E as DL-alpha-tocopheryl acetate, 2.2 mg vitamin K_3_ as menadione nicotinamide bisulfite, 27.6 μg vitamin B_12_, 4 mg riboflavin, 14 mg pantothenic acid as DL-calcium pantothenate, 30 mg niacin, 400 mg choline chloride, 0.7 mg folacin, 1.5 mg thiamin as thiamine mononitrate, 3 mg pyridoxine as pyridoxine hydrochloride, 44 μg biotin, 40 mg Mn as MnO, 75 mg Fe as FeSO_4_·H_2_O, 75 mg Zn as ZnO, 100 mg Cu as CuSO_4_·5H_2_O, 0.3 mg I as KI, and 0.3 mg Se as Na_2_SeO_3_.

**Table 4:** Effects of corn particle size on the apparent total tract digestibility of nutrients and the digestible energy of corn and diet fed to growing pigs

Items	Particle size (μm)					SEM	p-value		
	
	441	543	618	659	768		ANOVA	Linear	Quadratic
ATTD (%)									
Gross energy	90.6^a^	90.5^a^	89.4^ab^	88.6^bc^	87.6^c^	0.4	0.008	0.003	0.343
Crude protein	84.6	85.6	86.4	84.6	82.5	0.9	0.080	0.044	0.089
Ether extract	74.0^a^	56.9^b^	52.5^b^	41.1^c^	26.2^d^	2.5	0.001	0.009	0.012
Neutral detergent fiber	56.9^b^	61.4^ab^	67.0^a^	57.2^b^	54.8^b^	2.1	0.005	0.083	0.045
Acid detergent fiber	52.6^b^	58.5^ab^	63.8^a^	54.8^ab^	50.6^b^	2.2	0.009	0.078	0.013
Energy values (MJ/kg, as DM)									
Digestible energy of diet	16.3^a^	16.2^a^	16.0^ab^	15.9^bc^	15.7^c^	0.1	0.001	0.002	0.342
Digestible energy of corn	16.7^a^	16.7^a^	16.6^a^	16.5^ab^	16.3^b^	0.1	0.007	0.009	0.676

Data are means of six observations.

SEM, standard error of the mean; ANOVA, analysis of variance; ATTD, apparent total tract digestibility; DM, dry matter.

a–c Within a row means followed by the same letters are not different at p<0.05.

**Table 5:** Effect of corn lipid form on the apparent total tract digestibility of nutrients and the digestible energy (MJ/kg) of diets fed to growing pigs

Items	Experimental diets1)						SEM	p-value

	CG 2	CG 15	CO 1	CO 6	CO 1+CGM 2	CO 6+CGM 15		
ATTD (%)								
Gross energy	85.5^cd^	84.0^d^	89.5^a^	89.0^ab^	87.3^bc^	84.7^d^	0.5	0.010
Crude protein	79.5^bc^	81.4^ab^	84.6^a^	82.3^ab^	80.1^b^	75.5^c^	1.0	0.006
Ether extract	47.5^c^	47.9^c^	57.6^b^	75.0^a^	54.8^bc^	73.9^a^	1.8	0.009
Neutral detergent fiber	52.8^b^	64.2^a^	61.9^ab^	64.0^a^	58.7^ab^	62.7^ab^	2.4	0.046
Acid detergent fiber	39.2^b^	65.1^a^	57.3^ab^	59.5^a^	53.9^ab^	64.2^a^	4.1	0.001
Energy values (MJ/kg, as DM)								
Digestible energy of diet	15.7^d^	16.3^c^	16.40^bc^	17.2^a^	16.0^cd^	16.8^b^	0.1	0.004

Data are means of six observations.

SEM, standard error of the mean; ATTD, apparent total tract digestibility; DM dry matter.

1) CG 2 and CG 15, corn germ with inclusion rate of 2% and 15% in diet, respectively; CO 1+CGM 2, corn oil with inclusion rate of 1% combined with corn germ meal with inclusion rate of 2% in diet; CO 6+CGM 15, corn oil with inclusion rate of 6% combined with corn germ meal with inclusion rate of 15% in diet; CO 1 and CO 6, corn oil with inclusion rate of 1% and 6% in diet, respectively.

a–d Within a row means followed by the same letters are not different at p<0.05.
